# Molecular Characteristics and Genetic Diversity of Canine Parvovirus in Shanghai, China, from 2016 to 2025

**DOI:** 10.3390/microorganisms14040761

**Published:** 2026-03-27

**Authors:** Qiqi Xia, Jian Liu, Yaping Gui, Luming Xia, Chuangui Cao, Beijuan Chen, Xiangqian Yu, Weifeng Chen, Feng Xu, Jian Wang, Hongjin Zhao

**Affiliations:** 1 Shanghai Animal Disease Control Center, No. 30 Lane 855 Hongjing Road, Shanghai 201103, China; xiaqiqi1996@163.com (Q.X.); 13482222431@163.com (J.L.); 15800439747@163.com (Y.G.); xialuming@sina.com (L.X.); chenchenweifeng@163.com (W.C.); cmxf_16@163.com (F.X.); 2 Shanghai Jiading District Animal Disease Prevention and Control Center, Shanghai 201800, China; sallyccg@163.com; 3 Shanghai Baoshan District Animal Disease Prevention and Control Center, Shanghai 201900, China; 18930686603@163.com; 4 Shanghai Pudong New District Husbandry and Aquaculture Technology Extension Center, Shanghai 201299, China; yuxiangqian134@126.com

**Keywords:** canine parvovirus, CPV-2c, genotype shift, VP2, mutation

## Abstract

Canine parvovirus (CPV) is a major pathogen causing severe gastroenteritis in dogs. Since its emergence, CPV has undergone continuous evolution, leading to the predominance of variants such as CPV-2a, CPV-2b, and CPV-2c. To characterize the genetic features and evolutionary trends of CPV-2 at a regional level, 775 fecal samples were collected from domestic and stray dogs with suspected CPV-2 infection in Shanghai between 2016 and 2025. The overall positivity rate was 23.2% (180/775); incidence was substantially higher in stray dogs (30.2%) than in domestic dogs (15.9%). Thirty-one CPV-2 strains were successfully isolated. Temporal analysis revealed a pronounced genotype shift: isolates from 2016 to 2020 were predominantly New CPV-2a, whereas CPV-2c became the dominant genotype from 2021 through 2025. Sequence analysis identified the polymorphism of *VP2* gene and characteristic mutations F267Y, Y324I, N426E, Q370R and A440T in CPV-2c strains. A novel I447M mutation was detected in several isolates. Phylogenetic analysis showed that Shanghai isolates formed distinct clusters; CPV-2c strains were closely related to the Asian lineage. Structural modeling indicated that mutations at residues L87M, T101I, Y267F, A297S, G300A, Y305D, I324Y, Q370R, N426E, A440T, and I447M may alter the tertiary structure of the VP2 protein, potentially affecting antigenicity and receptor recognition. Collectively, these results demonstrate the complete genotype replacement of CPV-2 in Shanghai; CPV-2c is now predominant. Identification of the novel I447M mutation and structural analysis of key amino acid substitutions provide insight into CPV molecular evolution. These findings suggest that vaccines primarily based on older CPV-2 or CPV-2b genotypes offer suboptimal protection, highlighting the need for updated vaccine strategies targeting prevalent CPV-2c variants.

## 1. Introduction

Canine parvovirus (CPV) is an infectious disease that can cause severe vomiting, diarrhea, hemorrhagic gastroenteritis, and myocarditis [1,2]. Puppies aged 2–6 months are particularly susceptible, with high incidence and mortality rates [3]. CPV belongs to the family *Parvoviridae*, subfamily *Parvovirinae*, and genus *Protoparvovirus* [4]. Its 5.2-kb genome consists of single-stranded linear DNA [5]. The CPV genome encodes two structural proteins (VP1 and VP2) and two nonstructural proteins (NS1 and NS2) [6]. VP2 is the main capsid protein, constituting approximately 90% of the viral capsid [3,7]; it determines viral host range and plays a critical role in activating adaptive immune responses [8].

The evolution and transmission of CPV exhibit complex dynamics. Its single-stranded DNA genome confers a high mutation rate similar to that of RNA viruses [8], and mutations at key amino acid sites drive subtype replacement. CPV-2a, which emerged in 1979, replaced the original CPV-2; this was followed by mutations at residue 426 (N426D and N426E) in the *VP2* gene of CPV-2a, producing the CPV-2b and CPV-2c subtypes [3,9]. In recent years, CPV-2a and CPV-2b have diversified into new subtypes associated with the S297A mutation, resulting in pronounced antigenic differences [10]. Since the initial discovery of CPV in China in 1982, circulating strains have undergone continuous evolution from CPV-2a to CPV-2b and subsequently to CPV-2c, which has become the dominant genotype [11,12]. This trend is not unique to China, as the “Asian CPV-2c” variant has been identified in multiple countries [13,14,15]. In addition, CPV-2c has also become the main variant prevalent in Europe, CPV-2c has also frequently appeared in many European countries [16,17,18]. During viral evolution, key VP2 mutations such as N426D/E and S297A not only reshape host range (including infection of cats and other species) but also influence virulence and vaccine efficacy [19,20,21]. Continuous surveillance of genetic variation, development of targeted vaccines, and strengthening of integrated prevention and control measures are essential to limit CPV spread; its evolutionary patterns provide a valuable model for studies of viral adaptive evolution.

Shanghai is a major city in eastern China with over 24 million residents, and the numbers of stray and domestic dogs have greatly increased over the past decade [19]. Recent statistics indicate that the total number of pet dogs and cats in Shanghai has exceeded 2 million, with the number of pet-owning households reaching approximately 1.82 million, ranking among the highest in China [22]. Despite the implementation of routine vaccination that achieve relatively high coverage in some urban domestic dog populations, clinical cases of CPV infection continue to be observed annually in veterinary practices across the city, including in dogs with documented vaccination histories [23,24,25]. Given the widespread occurrence of canine parvovirus disease in both stray and domestic dogs—particularly the CPV-2c variant, which has become the dominant CPV-2 genotype in dogs in Shanghai and globally [26]—concerns have arisen regarding the protective efficacy of attenuated CPV-2 vaccines. Because stray dogs have extensive movement ranges and generally poor vaccination coverage, they are more susceptible to CPV-2 infection and dissemination. Here, we systematically investigated the prevalence of CPV-2 in domestic and stray dog populations in Shanghai over the past decade and analyzed the phylogenetic characteristics of viral mutations, with the aim of elucidating epidemic trends of CPV-2 strains and providing a foundation for molecular epidemiological research in this field.

## 2. Materials and Methods

### 2.1. Sample Collection and Viral DNA Extraction

**Figure 1:**
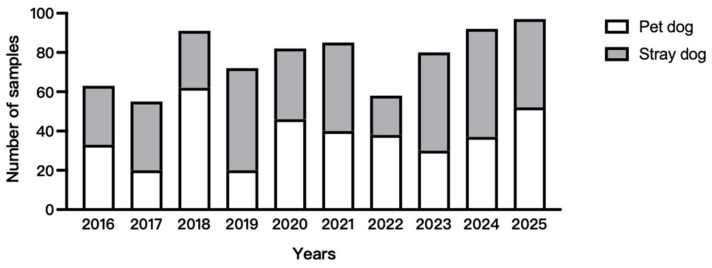
Collection of rectal swab samples from dogs with clinical gastroenteritis in Shanghai between 2016 and 2025. Gray represents the number of stray dogs, while white represents the number of pet dogs.

A total of 755 rectal swab samples were collected annually between 2016 and 2025 from dogs exhibiting clinical signs of gastroenteritis. Samples were obtained from two sources: (1) animal hospitals located in seven districts of Shanghai (Minhang, Baoshan, Pudong, Xuhui, Qingpu, Jiading, and Jing’an), covering the major urban and suburban areas of the city; and (2) stray dog shelters in Shanghai, which receive stray dogs from various districts across the city. This sampling strategy was designed to capture the genetic diversity of CPV circulating throughout the entire Shanghai area rather than focusing on a single facility or district. Annual sample numbers are shown in Figure 1, 378 samples were collected from pet dogs and 397 samples were collected from stray dogs. The sampling procedure was as follows: a cotton swab was soaked in sample processing solution, inserted approximately 2–3 cm into the dog’s anus, gently rotated to swab the anal mucosa, and then placed into a tube containing sample processing solution. All samples were stored at −80 °C. The Canine Parvovirus Colloidal Gold Test Strip (Nabai Biotech, Beijing, China; CAT: 010718898) was used to detect and screen positive samples. An appropriate amount of the sample was dispensed for testing using a plastic pipette, and 2–3 drops were slowly added into the sample well of the test strip. The results were observed after the sample had flowed through the window for 5 min, and reading was terminated at 15 min to determine the final result. The screened positive samples were inoculated into F81 cells, which were cultured in an incubator at 37 °C until cytopathic effects (CPE) were observed. The infected cells were then subjected to repeated freeze–thaw cycles to release viral particles. The supernatant was collected and stored at −80 °C for further analysis. Viral DNA was extracted from 50 µL of supernatant containing viral particles using the MagPure Virus RNA/DNA Assay Kit (Magen Biotech, Guangzhou, China; CAT: IVD5412). Extracted DNA was stored at −20 °C until further analysis.

### 2.2. Amplification and Sequencing of VP2

The CPV-2-specific primer pair VP2-F (5′-CGGGATCCATGAGTGATGGAGCAGTTCAA-3′) and VP2-R (5′-GGAATTCTTAGTATAATTTTCTAGGTGCTAGTT-3′) [27], synthesized by Shanghai Saiheng Biotechnology Co., Ltd. (Shanghai, China), was used to amplify the full-length *VP2* gene (1755 bp) by polymerase chain reaction (PCR) to confirm the presence of CPV-2 DNA [28]. To minimize the risk of contamination, standard precautions were strictly followed. All PCR setups were performed in a dedicated clean area physically separated from post-PCR handling, using aerosol-resistant filter tips and sterile aliquoted reagents. Each amplification run included a negative control (distilled water instead of template DNA) to monitor for potential cross-contamination. The amplification reactions were carried out in 50 μL PCR volume containing 25 μL of 2× Phanta Flash Master Mix (Vazyme Biotech, Nanjing, China; CAT: P510-01), 4 μL primers, 2 μL template, and 19 μL distilled water. Under the following cycling conditions: predenaturation at 95 °C for 5 min; 35 cycles of denaturation at 95 °C for 30 s, annealing at 56 °C for 30 s, and extension at 72 °C for 1 min; and a final extension at 72 °C for 7 min. Given the inherent limitations of Sanger sequencing in detecting low-abundance variants (<15–20%), we adopted a cloning-based sequencing strategy to enhance the reliability of genotype assignment. PCR products were cloned into the pMD™19-T Vector (Takara Biotech, Beijing, China; CAT: 6013), and each amplicon was subjected to Sanger dideoxy sequencing (Shanghai Saiheng Biotechnology Co., Ltd., Shanghai, China).

### 2.3. Sequence Analysis

VP2 sequence amplified from the CPV-2 strain DNA isolated from specimens were aligned with those of 44 reference CPV strains (Table 1) using the ClustalW algorithm implemented in MEGA 7.0 software (https://www.megasoftware.net (accessed on 28 October 2025)). Nucleotide and amino acid identity analyses were subsequently conducted.

### 2.4. Phylogenetic Analysis

To assess the genetic diversity of CPV isolates from Shanghai, VP2 nucleotide sequences from 31 strains were compared with those from 44 reference strains. Reference sequences representing all major CPV genotypes (CPV-2, CPV-2a, CPV-2b, CPV-2c, New CPV-2a, New CPV-2b, and FPV) were selected from the GenBank database based on previously published phylogenetic studies [19,27,29,30], ensuring the inclusion of well-characterized prototype strains for each genotype. These sequences encompass diverse geographical origins and temporal ranges to provide a comprehensive framework for phylogenetic comparison. Phylogenetic trees based on VP2 amino acid sequences were constructed by the maximum-likelihood method with FLU + I as the best-fit model implemented in MEGA 7.0 software (https://www.megasoftware.net), with 1000 bootstrap replicates [29].

### 2.5. Tertiary Structure Prediction

The tertiary structure of the VP2 protein was predicted using the SWISS-MODEL server (https://swissmodel.expasy.org (accessed on 22 December 2025)) [31]. The predicted models were visualized in PyMOL 3.1 software (https://pymol.org (accessed on 23 December 2025)) [31]; amino acid substitution sites were mapped onto the VP2 structure to determine their locations and potential effects on protein conformation.

## 3. Results

### 3.1. Isolation and Genotyping of CPV-2

In total, 775 rectal swab samples from dogs with suspected CPV-2 infection were collected in Shanghai between 2016 and 2025. CPV positive screening was performed using the Canine Parvovirus Colloidal Gold Test Strip. Among these samples, 180 showed positive test results, yielding an overall positivity rate of 23.2% (180/775). Positivity rates were 15.9% (60/378) in domestic dogs and 30.2% (120/397) in stray dogs. Annual CPV-2 positivity rates for rectal swab samples are summarized in Table 2. After isolation and propagation of positive samples in F81 cells, 31 CPV-2 strains exhibiting typical cytopathic effects were obtained: 20 from stray dogs and 11 from pet dogs. Table 3 provides detailed information on sampling date, location, clinical signs, gross lesions, and CPV-2 immunization status. For the purposes of this study, immunization status was recorded based on owner-reported vaccination history or veterinary medical records at the time of sample collection. Dogs were classified as “Yes” if they had received at least one dose of a CPV-2-containing vaccine within the year prior to the onset of clinical signs. Dogs were classified as “No” if there was no documented history of vaccination or if the owner confirmed that the dog had never been vaccinated.

### 3.2. Amino Acid Mutation Analysis of VP2

The *VP2* genes of the 31 CPV-2 isolates were amplified by PCR and sequenced. To identify amino acid substitutions in *VP2*, we compared sequences from these isolates with the sequences of 20 reference strains (Table 4). Sixteen isolates (SH2019-1, SH2020-3, SH2021-1, SH2021-2, SH2021-3, SH2022-1, SH2022-3, SH2023-1, SH2023-2, SH2024-1, SH2024-2, SH2024-3, SH2025-1, SH2025-2, SH2025-3, and SH2025-4) were classified as CPV-2c; they exhibited the characteristic amino acid substitutions Q370R, N426E, and A440T. The I447M substitution was detected in isolates SH2021-1, SH2023-3, SH2024-1, and SH2025-2. The SH2023-1 isolate contained key substitutions at positions L87M, T101I, G300A, and Y305D, consistent with the CPV-2 genotype; it also harbored mutations at Y267F, A297S, I324Y, and A440T. The remaining 14 isolates were classified as New CPV-2a and displayed the T440A substitution.

**Figure 2:**
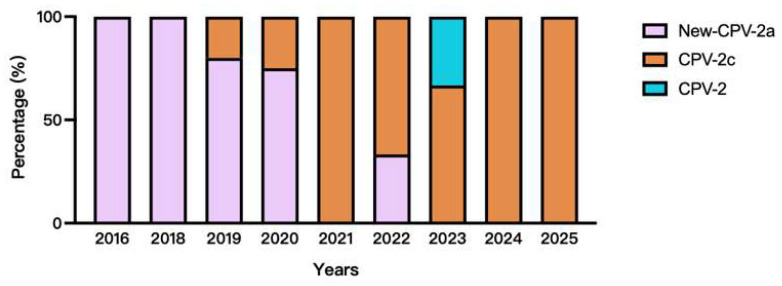
The isolation status of CPV positive strains and the proportion of different genotypes. Pink represents the New CPV-2a genotype, orange represents the CPV-2c genotype, and blue represents the CPV-2 genotype.

Next, we analyzed genotype changes in CPV-2 strains isolated between 2016 and 2025 (Figure 2). All CPV isolates obtained in 2016 and 2018 were classified as New CPV-2a. CPV-2c strains first appeared in 2019; however, most isolates from 2019 and 2020 remained New CPV-2a. Beginning in 2021, a clear genotype shift was observed, with a pronounced increase in CPV-2c strains over the subsequent 5 years. All isolates obtained in 2021, 2024, and 2025 belonged to the CPV-2c genotype; in 2023, a single CPV-2 isolate with a sequence similar to the vaccine strain was identified.

### 3.3. Geographic Distribution of CPV Genotypes Across Shanghai Districts

To assess whether this shift represented a citywide phenomenon, we examined the geographic distribution of genotypes across the seven sampled districts (Table 5). In Minhang, CPV-2c appeared as early as 2019–2020 and progressively replaced New CPV-2a, becoming the major genotype by 2024–2025. In Pudong, Xuhui, and Baoshan, New CPV-2a predominated until 2020, after which CPV-2c became dominant in 2021–2023 and exclusively detected in 2024–2025. Qingpu and Jing’an, though lacking early isolates, showed CPV-2c as the major genotype once sampling commenced in 2019–2020 and 2021–2023, respectively. Jiading had only New CPV-2a isolates in early years and no later isolates. Overall, CPV-2c emerged and became predominant in all districts with sufficient longitudinal sampling, and the timing of the shift was consistent across locations. These spatial data, combined with the temporal trend, demonstrate that the major genotype shift from New CPV-2a to CPV-2c occurred broadly across Shanghai rather than being confined to a single facility or district.

### 3.4. Phylogenetic Analysis of the VP2 Gene

**Figure 3:**
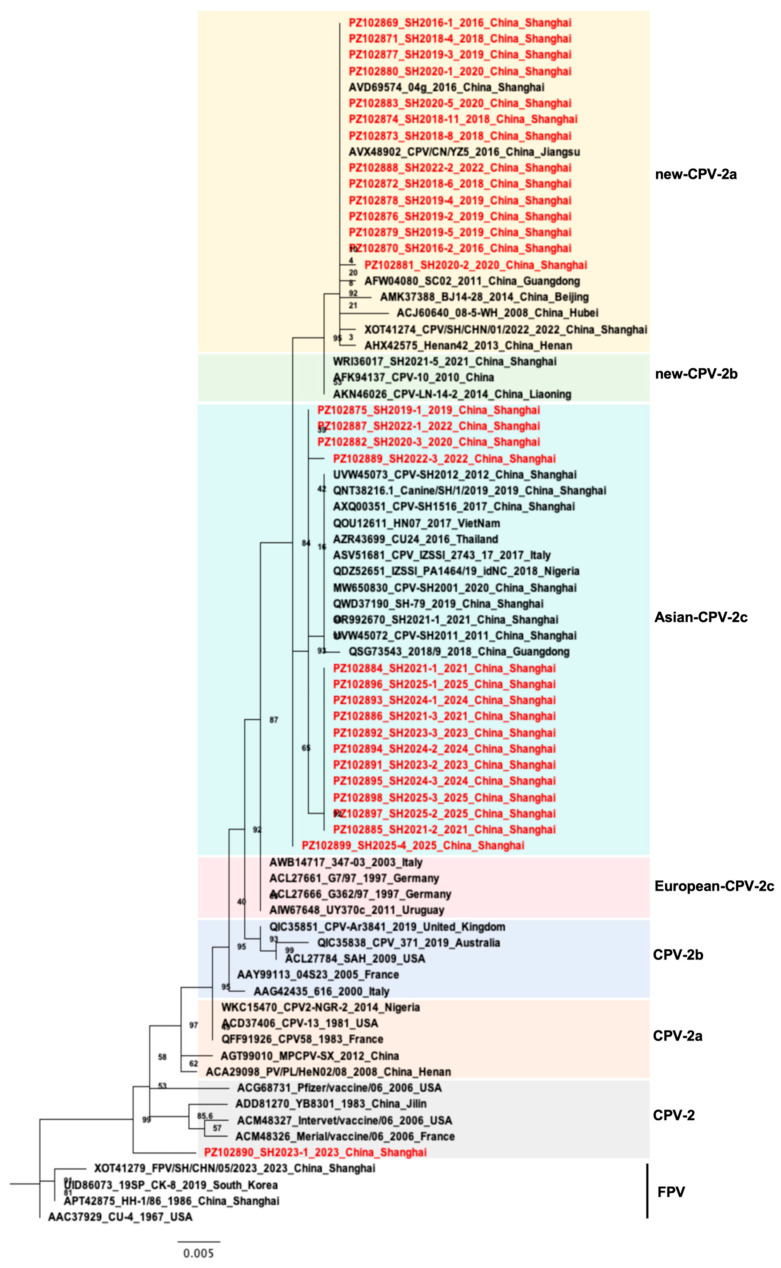
Phylogenetic tree analysis. A phylogenetic tree based on 31 *VP2* gene sequences of isolates and 44 reference FPV/CPV strains was constructed by the maximum likelihood method using the MEGA 7.0 software, and evolutionary distances were determined based on the FLU + I matrix-based model, with 1000 boot strap replicates. The new CPV isolates identified in this study are marked with red.

Forty-four CPV reference strains representing different genotypes were retrieved from the GenBank database. Phylogenetic trees were constructed based on VP2 amino acid sequences from the 31 CPV isolates, together with the reference strains. The analysis showed that one isolate (SH2023-1) clustered within the CPV-2 branch. Sixteen isolates from 2019 to 2025 were grouped within the Asian CPV-2c lineage and showed close links to CPV strains of Asian origin (Figure 3). The remaining 14 isolates, collected in 2016, 2018, 2019, 2020, and 2022, clustered within the New CPV-2a branch; they were closely related to strains identified in Shanghai, Guangdong, Beijing, Hubei, Henan, and other regions of China.

### 3.5. Tertiary Structure Analysis of the VP2 Protein

**Figure 4:**
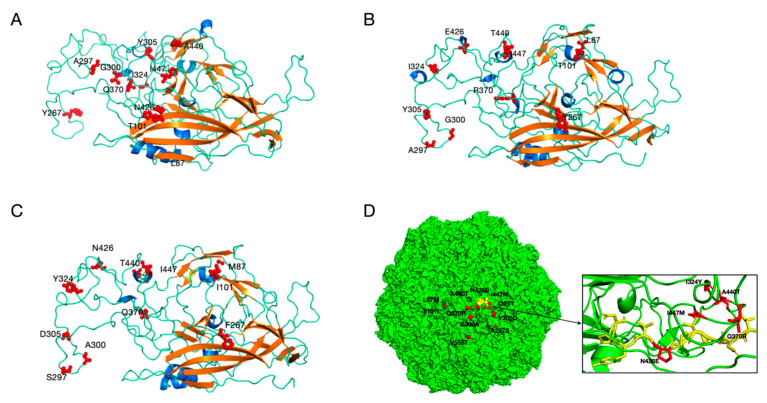
Predicted tertiary structures of VP2 protein from three CPV genotypes detected in Shanghai. (**A**) New CPV-2a (PDB ID code 9E8D). (**B**) CPV-2c (PDB ID code 7UTU). (**C**) CPV-2 (PDB ID code 2CAS). Surface-exposed residues that differ between genotypes are highlighted in red. α-helices are shown in dark blue, β-sheets in orange, and random coils in light blue. (**D**) The structural model of CPV-2, with mutated amino acid sites represented in red and GH loop represented in yellow.

To visualize the spatial distribution of amino acid substitutions identified in this study, homology models of the VP2 protein were constructed for the three CPV genotypes detected in Shanghai (New CPV-2a, CPV-2c, and CPV-2) using the SWISS-MODEL server. Different template structures were employed to ensure optimal homology modeling for each genotype, thereby providing the most accurate structural context for interpreting mutations in these strains. Structural mapping (Figure 4) revealed that the majority of mutated residues identified in Shanghai isolates—including L87M, T101I, Y267F, A297S, G300A, Y305D, I324Y, Q370R, N426E, A440T, and the novel I447M substitution—are located on surface-exposed regions of the viral capsid. These structural mapping suggests that the observed substitutions are not randomly distributed but are concentrated in regions critical for receptor recognition, may affect the tertiary structure of the VP2 protein. Notably, residue 426 (N426E in CPV-2c) is situated in the GH loop of VP2, a structural element known to form part of the receptor-binding interface. The I447M substitution is located in the C-terminal region of VP2; in the folded protein structure, residues 426 and 447 are in close spatial proximity. Other substitutions, such as Y267F and Y324I, are located in the β-barrel core of the protein.

## 4. Discussion

In recent years, rapid expansion of the pet industry has led to an increase in companion dog populations, highlighting the need to address emerging infectious diseases [12]. Canine parvovirus–associated diarrhea, a severe threat to canids, has attracted considerable attention. CPV-2 was initially detected in China in the early 1980s, and CPV-2a became the predominant genotype by 1986 [19]. Since 2010, CPV-2c has been increasingly detected in eastern, northern, northeastern, and southern regions of China, aligning with its global dissemination and current predominance [32,33]. This pattern underscores the ongoing evolution and strong regional transmissibility of canine parvovirus.

Our data show a substantial difference in infection rates between stray and domestic dogs; the positivity rate was higher in stray dogs (30.2%) than in pet dogs (15.9%). This disparity in infection rates can be partly attributed to the distinct living environments of these two populations in Shanghai. The majority of pet dogs in this metropolitan area are primarily kept indoors, with limited exposure to uncontrolled external environments and potentially infected animals. In contrast, stray dogs inhabit outdoor environments characterized by high population density and lack of sanitary oversight. Furthermore, the urban landscape of Shanghai facilitates frequent indirect and direct contact between stray and pet dog populations. During outdoor activities such as walks in public parks or residential areas, pet dogs may encounter stray dogs or come into contact with environments contaminated by them (e.g., feces, soil). This ecological interface serves as a critical bridge for pathogen spillover, allowing the high viral burden circulating in stray populations to pose a continuous infection risk to household pets. This observation is consistent with the limited vaccination coverage and uncontrolled living conditions of stray dogs, which serve as the main hosts and drivers of CPV-2 transmission and evolution. Although most stray dogs do not experience vaccine induced selection pressure, gene mutations and genotype changes still occur, which may be due to the high population density and continuous transmission chains among stray dogs provide extensive opportunities for viral replication and mutation accumulation, effectively fueling viral evolution. In addition, natural selection acts on viral fitness traits such as transmission efficiency, environmental stability, and receptor binding. CPV-2c may possess intrinsic replication advantages or enhanced environmental persistence that facilitate its spread in high-density stray populations independent of immune evasion. Futhermore, genetic drift may play a role. Thus, while vaccination influences viral evolution in domestic dogs, the stray dog reservoir functions as an independent evolutionary arena where viral diversity emerges and is maintained through sustained transmission. The high genetic diversity within these populations may facilitate the emergence of novel variants. All dogs included in this study presented with clinical signs consistent with canine parvovirus infection, including lethargy, anorexia, vomiting, and hemorrhagic diarrhea of varying severity. The majority of cases exhibited moderate to severe clinical manifestations requiring veterinary intervention. However, due to the retrospective nature of sample collection and the lack of standardized clinical scoring at the time of presentation, we were unable to perform a quantitative correlation between specific clinical signs and the infecting CPV genotype. Nevertheless, no obvious differences in clinical presentation were observed between dogs infected with CPV-2c versus those infected with New CPV-2a, suggesting that the ongoing genotype shift may not be associated with a discernible change in disease phenotype.

The geographic stratification of our isolates further supports the interpretation that the CPV-2c genotype shift is a citywide phenomenon rather than a localized outbreak. CPV-2c strains were detected in samples collected from veterinary hospitals and shelters across all seven surveyed districts, with the transition from New CPV-2a to CPV-2c occurring synchronously in multiple locations between 2019 and 2021. This pattern is inconsistent with a single-facility outbreak and instead reflects broader epidemiological dynamics operating at the metropolitan scale. Although sample sizes from individual districts varied annually, the consistency of the genotypic shift across geographically distinct sampling sites provides empirical evidence for the widespread dissemination of CPV-2c throughout Shanghai. The rapid expansion of Asian-derived CPV-2c in China suggests the presence of evolutionary advantages [34]. Given the role of *VP2* as a surface structural protein that regulates host tropism [35,36], hemagglutination properties, and antigenicity through induction of neutralizing antibody responses, we sequenced *VP2* genes from rectal swabs of dogs that exhibited gastroenteritis in Shanghai between 2016 and 2025. Most CPV-2c isolates harbored characteristic substitutions (F267Y, Y324I, Q370R, N426E, and A440T), consistent with previously reported strains from Shanghai and Beijing [26,37]. Additionally, a unique I447M substitution was identified in some isolates. The F267Y, Y324I, N426E, and A440T substitutions have been identified as predominant mutations during early stages of CPV evolution [9,38]. Residues 267 and 426 are considered critical determinants of antigenicity and host receptor recognition. The N426E substitution, a defining feature of CPV-2c, is located in the GH loop of VP2 and enhances binding affinity to the canine transferrin receptor, which may explain the increased fitness of this variant. The Q370R substitution was initially identified in CPV-2a strains isolated from giant pandas in Sichuan Province, China, and subsequently became prevalent in CPV-2c strains [39]. The functional significance of the I447M substitution identified in this study remains unclear. A key finding of this study is the temporal shift in genotype predominance. Although New CPV-2a was predominant between 2016 and 2020, a clear transition occurred in 2021, culminating in complete dominance of CPV-2c by 2025, consistent with a report by Liu et al. [26]. Phylogenetic analysis confirmed that the CPV-2c isolates clustered closely with previously reported Asian CPV-2c strains from Shanghai, forming distinct lineages separate from European and American epidemic strains. This rapid replacement suggests that CPV-2c possesses substantial adaptive advantages over earlier variants in the Shanghai ecosystem. The observed major genotypic shift towards CPV-2c predominance, particularly within the unvaccinated stray dog population, rather than immune evasion, is the primary driver of selection. CPV-2c, with its enhanced binding affinity to the canine transferrin receptor (conferred by the N426E substitution), likely possesses a transmission advantage in dense, immunologically naïve populations, allowing it to outcompete older variants like New CPV-2a.

Previous studies have shown that residues 267 and 426, which exhibit high mutation frequencies in CPV-2, play important antigenic roles and influence the tertiary structure of VP2 [40]. The S297A substitution—detected in all strains in the present study—has been reported to induce antigenic changes in CPV-2 variants and may substantially contribute to ongoing host adaptation [41]. Notably, many of the mutated residues identified in our Shanghai isolates (including S297A, G300A, I324Y, Q370R, and N426E) are located within or adjacent to known B-cell epitopes, such as loop 3 (residues 298–302) and the GH loop (residues 420–430), which are critical targets for neutralizing antibodies [42,43,44]. Pan et al. predicted that substitutions at positions M87L, I101T, S297A, and N426E alter the surface topology of the viral capsid, potentially affecting antibody neutralization [27]. A critical finding of our structural analysis is the spatial clustering of mutations around the receptor-binding interface. Residue 426, located in the GH loop, has been conclusively shown by X-ray crystallography and mutagenesis studies to be a key determinant of canine transferrin receptor (TfR) recognition. The N426E substitution characteristic of CPV-2c enhances binding affinity by creating favorable electrostatic interactions with positively charged residues (e.g., arginine and lysine) on TfR, as demonstrated in the structural study by Lee et al. [31]. Our models confirm that this substitution introduces a negatively charged side chain at a position that directly contacts the receptor in the viral-receptor complex. Additionally, residue 447 is located in the C-terminal region of *VP2*, adjacent to key functional domains, including the receptor-binding region containing residue 426. Its potential synergistic interaction with N426E may enhance viral affinity for the canine transferrin receptor. I447M may exert a synergistic effect on receptor binding by stabilizing the conformation of the GH loop or by contributing to the local electrostatic environment. The substitution of isoleucine (a hydrophobic residue with a branched side chain) to methionine (a longer, sulfur-containing, flexible side chain) could alter loop dynamics or create additional van der Waals contacts with neighboring residues, potentially fine-tuning the receptor-binding interface. Future studies employing site-directed mutagenesis and surface plasmon resonance (SPR) binding assays are needed to quantitatively assess the impact of I447M, both alone and in combination with N426E, on TfR binding affinity. Accordingly, our analyses predicted that substitutions at positions L87M, T101I, Y267F, A297S, G300A, Y305D, I324Y, Q370R, N426E, A440T, and I447M alter the tertiary structure of the VP2 protein. These structural changes may result in altered antigenicity and modified cellular receptor recognition, and further experiments are needed for verification.

Although vaccination is the primary strategy for CPV prevention in China, CPV-2c breakthrough infections persist [3,20,21,45]. Vaccine failure may be associated with maternal antibody interference [46,47,48]. Notably, vaccines currently used in China primarily target ancestral CPV-2 or CPV-2b antigens [38] and may offer limited protection against CPV-2c [3,24,49]. Here, we found that domestic dogs with a history of CPV vaccination were still infected, implying that existing vaccines have insufficient protective effects. Long-term surveillance of viral mutations and regional prevalence is essential to guide the development of CPV-2c-specific vaccines. This study has several limitations that should be acknowledged. First, our sampling was restricted to dogs presenting with clinical signs of gastroenteritis; we did not screen asymptomatic or subclinically infected dogs, which may serve as unrecognized reservoirs for CPV transmission. The prevalence and genetic characteristics of CPV in asymptomatic carriers remain unknown and warrant future investigation. Recent studies in healthy dog populations have demonstrated that parvovirus shedding occurs in apparently healthy animals. Ferrara et al. investigated 170 apparently healthy dogs in Italy and detected CPV-2 in 6.5% (11/117) of fecal samples using real-time PCR, confirming that asymptomatic carriers actively shed the virus [4]. Importantly, their risk factor analysis revealed significantly higher parvovirus detection rates in animals of stray origin, those with altered fecal scores, and those living outdoors. These findings align with our observation of higher infection rates in stray dogs and underscore the importance of asymptomatic carriers in maintaining viral circulation within dog populations. Second, although our phylogenetic analysis clearly demonstrates clustering of Shanghai CPV-2c isolates with Asian strains, the limited number of European and American reference sequences included in this study precludes a comprehensive global phylogeographic analysis. Third, while we identified a novel I447M substitution and predicted its potential structural impact using in silico modeling, these predictions remain speculative in the absence of functional validation. No in vitro or in vivo experiments were conducted to confirm the effects of this mutation—or the other identified substitutions—on viral antigenicity, receptor binding affinity, or virulence. Future studies employing reverse genetics systems to engineer these mutations into infectious clones, followed by in vitro receptor binding assays and animal challenge studies, are essential to establish causality. Despite these limitations, this study provides a decade-long (2016–2025) overview of CPV genotypes in Shanghai, characterizes emerging mutant strains and novel substitutions, and establishes a theoretical basis for improving CPV-2c vaccine strategies and reducing disease burden.

## 5. Conclusions

In this study, we collected 775 fecal samples from dogs with suspected CPV-2 infection, including both domestic and stray dogs, in Shanghai between 2016 and 2025; we successfully isolated 31 CPV strains. Genotype analysis stratified by sampling location revealed that the shift from New CPV-2a to CPV-2c occurred consistently across multiple districts, with CPV-2c becoming the predominant genotype citywide from 2021 onward. The CPV-2c strains identified in this study harbored the characteristic substitutions F267Y, Y324I, N426E, and A440T, as well as a novel I447M substitution; its functional significance warrants further investigation. These findings suggest that CPV-2c continues to undergo evolutionary change and that vaccines developed against CPV-2 or CPV-2b genotypes may not provide complete protection. Therefore, this decade-long surveillance of CPV strains in Shanghai provides a theoretical basis for the development of improved vaccines.

## Figures and Tables

**Table 1:** Information about CPV reference strains.

Genetype	Genbank No.	Strain	Year	Country (Province)
CPV-2b	QIC35851	CPV-Ar3841	2019	United Kingdom
CPV-2b	AAG42435	616	2000	Italy
CPV-2b	AAY99113	04S23	2005	France
CPV-2b	ACL27784	SAH	2009	USA
CPV-2b	QIC35838	CPV_371	2019	Australia
Asian-CPV-2c	QNT38216.1	Canine:SH:1:2019	2019	China, Shanghai
Asian-CPV-2c	QOU12611	HN07	2017	Viet Nam
Asian-CPV-2c	QWD37190	SH-79	2019	China, Shanghai
Asian-CPV-2c	MW650830	CPV-SH2001	2020	China, Shanghai
Asian-CPV-2c	UVW45073	CPV-SH2012	2012	China, Shanghai
Asian-CPV-2c	AZR43699	CU24	2016	Thailand
Asian-CPV-2c	AXQ00351	CPV-SH1516	2017	China, Shanghai.
Asian-CPV-2c	QDZ52651	IZSSI_PA1464:19_idNC	2018	Nigeria
Asian-CPV-2c	QSG73543	2018:09:00	2018	China, Guangdong
Asian-CPV-2c	ASV51681	CPV_IZSSI_2743_17	2017	Italy
Asian-CPV-2c	UVW45072	CPV-SH2011	2011	China, Shanghai
Asian-CPV-2c	OR992670	SH2021-1	2021	China, Shanghai
FPV	UID86073	19SP_CK-8	2019	South Korea
FPV	XOT41279	FPV:SH:CHN:05:2023	2023	China, Shanghai
FPV	AAC37929	CU-4	1967	USA
FPV	APT42875	HH-1:86	1986	China, Shanghai
CPV-2	ACM48326	Merial:vaccine:06	2006	France
CPV-2	ADD81270	YB8301	1983	China, Jilin
CPV-2	ACM48327	Intervet:vaccine:06	2006	USA
CPV-2	ACG68731	Pfizer:vaccine:06	2006	USA
new-CPV-2a	AFW04080	SC02	2011	China, Guangdong
new-CPV-2a	AVD69574	04g	2016	China, Shanghai
new-CPV-2a	AHX42575	Henan42	2013	China, Henan
new-CPV-2a	XOT41274	CPV:SH:CHN:01:2022	2022	China, Shanghai
new-CPV-2a	AMK37388	BJ14-28	2014	China, Beijing
new-CPV-2a	AVX48902	CPV:CN:YZ5	2016	China, Jiangsu
new-CPV-2a	ACJ60640	08-5-WH	2008	China, Hubei
CPV-2a	ACD37406	CPV-13	1981	USA
CPV-2a	ACA29098	PV:PL:HeN02:08	2008	China, Henan
CPV-2a	QFF91926	CPV58	1983	France
CPV-2a	WKC15470	CPV2-NGR-2	2014	Nigeria
CPV-2a	AGT99010	MPCPV-SX	2012	China
European-CPV-2c	AIW67648	UY370c	2011	Uruguay
European-CPV-2c	ACL27666	G362:97	1997	Germany
European-CPV-2c	ACL27661	G7:97	1997	Germany
European-CPV-2c	AWB14717	347-03	2003	Italy
new-CPV-2b	AKN46026	CPV-LN-14-2	2014	China, Liaoning
new-CPV-2b	AFK94137	CPV-10	2010	China
new-CPV-2b	WRI36017	SH2021-5	2021	China, Shanghai

**Table 2:** CPV positivity rates in dog fecal samples (No. positive samples/No. samples).

Source	Total	2016	2017	2018	2019	2020	2021	2022	2023	2024	2025
Pet dog	15.9% (60/378)	15.1% (5/33)	10% (2/20)	14.5% (9/62)	15% (3/20)	10.9% (5/46)	20% (8/40)	13.2% (5/38)	30% (9/30)	24.3% (9/37)	9.6% (5/52)
Stray dog	30.2% (120/397)	16.7% (5/30)	11.4% (4/35)	17.2% (5/29)	32.7% (17/52)	52.8% (19/36)	26.7% (12/45)	45% (9/20)	50% (25/50)	27.3% (15/55)	20% (9/45)

**Table 3:** Summary of CPV-2 isolates from dogs in Shanghai, China.

Number	Strain	Genbank No.	Year	Genetype	Vaccinate	Source	District
1	SH2016-1	PZ102869	2016	New CPV-2a	No	Stray dog	Minhang
2	SH2016-2	PZ102870		New CPV-2a	No	Stray dog	Xuhui
3	SH2018-4	PZ102871	2018	New CPV-2a	Yes	Pet dog	Pudong
4	SH2018-6	PZ102872		New CPV-2a	Yes	Pet dog	Pudong
5	SH2018-8	PZ102873		New CPV-2a	Yes	Pet dog	Jiading
6	SH2018-11	PZ102874		New CPV-2a	Yes	Pet dog	Baoshan
7	SH2019-1	PZ102875	2019	CPV-2c	No	Stray dog	Minhang
8	SH2019-2	PZ102876		New CPV-2a	No	Stray dog	Minhang
9	SH2019-3	PZ102877		New CPV-2a	No	Stray dog	Pudong
10	SH2019-4	PZ102878		New CPV-2a	No	Stray dog	Xuhui
11	SH2019-5	PZ102879		New CPV-2a	No	Stray dog	Xuhui
12	SH2020-1	PZ102880	2020	New CPV-2a	No	Stray dog	Jiading
13	SH2020-2	PZ102881		New CPV-2a	No	Stray dog	Qingpu
14	SH2020-3	PZ102882		CPV-2c	No	Stray dog	Minhang
15	SH2020-5	PZ102883		New CPV-2a	No	Stray dog	Baoshan
16	SH2021-1	PZ102884	2021	CPV-2c	Yes	Pet dog	Minhang
17	SH2021-2	PZ102885		CPV-2c	Yes	Pet dog	Minhang
18	SH2021-3	PZ102886		CPV-2c	No	Stray dog	Jiading
19	SH2022-1	PZ102887	2022	CPV-2c	No	Stray dog	Jing’an
20	SH2022-2	PZ102888		New CPV-2a	No	Stray dog	Minhang
21	SH2022-3	PZ102889		CPV-2c	No	Stray dog	Pudong
22	SH2023-1	PZ102890	2023	CPV-2	Yes	Pet dog	Baoshan
23	SH2023-2	PZ102891		CPV-2c	Yes	Pet dog	Baoshan
24	SH2023-3	PZ102892		CPV-2c	No	Stray dog	Xuhui
25	SH2024-1	PZ102893	2024	CPV-2c	Yes	Pet dog	Minhang
26	SH2024-2	PZ102894		CPV-2c	No	Stray dog	Xuhui
27	SH2024-3	PZ102895		CPV-2c	No	Stray dog	Jing’an
28	SH2025-1	PZ102896	2025	CPV-2c	Yes	Pet dog	Pudong
29	SH2025-2	PZ102897		CPV-2c	Yes	Pet dog	Baoshan
30	SH2025-3	PZ102898		CPV-2c	No	Stray dog	Qingpu
31	SH2025-4	PZ102899		CPV-2c	No	Stray dog	Qingpu

**Table 4:** Amino acid substitutions in the VP2 region of CPV-2 strains identified in this study compared with reference strains.

Strain	Year	Genetype	Orign	5	87	101	267	297	300	305	324	370	426	440	447	555
YB8301	1983	CPV-2	China, Jilin	A	M	I	F	S	D	D	Y	Q	N	T	I	V
QHD	2019		China	A	M	I	F	A	A	D	Y	Q	N	T	I	V
06	2006		USA	A	M	I	F	S	A	D	Y	Q	N	T	I	V
388/05-3	2005		Italy	A	M	I	F	S	A	D	Y	Q	N	T	I	V
**SH2023-1**	2023		China, Shanghai	A	M	I	F	S	A	D	Y	Q	N	T	I	V
Canine/SH/1/2019	2019	CPV-2c	China, Shanghai	G	L	T	Y	A	G	Y	I	R	E	T	I	V
SH2001	2020		China, Shanghai	G	L	T	Y	A	G	Y	I	R	E	T	I	V
SH1516	2017		China, Shanghai	G	L	T	Y	A	G	Y	I	R	E	T	I	V
SH2011	2011		China, Shanghai	G	L	T	Y	A	G	Y	I	R	E	T	I	V
CU24	2016		Thailand	G	L	T	Y	A	G	Y	I	R	E	T	I	V
**SH2019-1**	2019		China, Shanghai	A	L	T	Y	A	G	Y	I	R	E	T	I	V
**SH2020-3**	2020		China, Shanghai	A	L	T	Y	A	G	Y	I	R	E	T	I	V
**SH2021-1**	2021		China, Shanghai	A	L	T	Y	A	G	Y	I	R	E	T	M	V
**SH2021-2**	2021		China, Shanghai	A	L	T	Y	A	G	Y	I	R	E	T	I	V
**SH2021-3**	2021		China, Shanghai	A	L	T	Y	A	G	Y	I	R	E	T	I	V
**SH2022-1**	2022		China, Shanghai	A	L	T	Y	A	G	Y	I	R	E	T	I	V
**SH2022-3**	2022		China, Shanghai	A	L	T	Y	A	G	Y	I	R	E	T	I	V
**SH2023-2**	2023		China, Shanghai	A	L	T	Y	A	G	Y	I	R	E	T	M	V
**SH2023-3**	2023		China, Shanghai	A	L	T	Y	A	G	Y	I	R	E	T	I	V
**SH2024-1**	2024		China, Shanghai	A	L	T	Y	A	G	Y	I	R	E	T	M	V
**SH2024-2**	2024		China, Shanghai	A	L	T	Y	A	G	Y	I	R	E	T	I	V
**SH2024-3**	2024		China, Shanghai	A	L	T	Y	A	G	Y	I	R	E	T	I	V
**SH2025-1**	2025		China, Shanghai	A	L	T	Y	A	G	Y	I	R	E	T	I	V
**SH2025-2**	2025		China, Shanghai	A	L	T	Y	A	G	Y	I	R	E	T	M	V
**SH2025-3**	2025		China, Shanghai	A	L	T	Y	A	G	Y	I	R	E	T	I	V
**SH2025-4**	2025		China, Shanghai	A	L	T	Y	A	G	Y	I	Q	E	T	I	V
04g	2016	New CPV-2a	China, Shanghai	A	L	T	Y	A	G	Y	I	Q	N	A	I	V
Henan42	2013		China, Henan	A	L	T	Y	A	G	Y	I	Q	N	A	I	V
CPV/SH/CHN/01/2022	2022		China, Shanghai	A	L	T	Y	A	G	Y	I	Q	N	A	I	V
08-5-WH	2008		China, Hubei	A	L	T	Y	A	G	Y	I	Q	N	A	I	V
**SH2016-1**	2016		China, Shanghai	A	L	T	Y	A	G	Y	I	Q	N	A	I	V
**SH2016-2**	2016		China, Shanghai	A	L	T	Y	A	G	Y	I	Q	N	A	I	V
**SH2018-4**	2018		China, Shanghai	A	L	T	Y	A	G	Y	I	Q	N	A	I	V
**SH2018-6**	2018		China, Shanghai	A	L	T	Y	A	G	Y	I	Q	N	A	I	V
**SH2018-8**	2018		China, Shanghai	A	L	T	Y	A	G	Y	I	Q	N	A	I	V
**SH2018-11**	2018		China, Shanghai	A	L	T	Y	A	G	Y	I	Q	N	A	I	V
**SH2019-2**	2019		China, Shanghai	A	L	T	Y	A	G	Y	I	Q	N	A	I	V
**SH2019-3**	2019		China, Shanghai	A	L	T	Y	A	G	Y	I	Q	N	A	I	V
**SH2019-4**	2019		China, Shanghai	A	L	T	Y	A	G	Y	I	Q	N	A	I	V
**SH2019-5**	2019		China, Shanghai	A	L	T	Y	A	G	Y	I	Q	N	A	I	V
**SH2020-1**	2020		China, Shanghai	A	L	T	Y	A	G	Y	I	Q	N	A	I	V
**SH2020-2**	2020		China, Shanghai	A	L	T	Y	A	G	Y	I	Q	N	A	I	V
**SH2020-5**	2020		China, Shanghai	A	L	T	Y	A	G	Y	I	Q	N	A	I	V
**SH2022-2**	2022		China, Shanghai	A	L	T	Y	A	G	Y	I	Q	N	A	I	V
CPV-LN-14-2	2014	New CPV-2b	China, Liaoning	A	L	T	Y	A	G	Y	I	Q	D	A	I	V
SH2021-5	2021		China, Shanghai	A	L	T	Y	A	G	Y	I	Q	D	A	I	V
PV/PL/HeN02/08	2008	CPV-2a	China, Henan	A	L	T	F	S	A	D	Y	Q	N	T	I	V
CPV58	1983		France	A	L	T	F	S	G	Y	Y	Q	N	T	I	V
616	2000	CPV-2b	Italy	A	L	T	F	S	G	Y	Y	Q	D	T	I	V
SAH	2009		USA	A	L	T	F	A	G	Y	Y	Q	D	T	I	V
CPV-Ar3841	2019		United Kingdom	A	L	T	F	A	G	Y	Y	Q	D	T	I	V

The bold text indicates the isolated strains in this study.

**Table 5:** Distribution of CPV genotypes by district and year.

District	2016–2018	2019–2020	2021–2023	2024–2025	Major Genotypes Shift Observed
Minhang	New CPV-2a	New CPV-2a/CPV-2c	New CPV-2a/CPV-2c	CPV-2c	Yes
Pudong	New CPV-2a	New CPV-2a	CPV-2c	CPV-2c	Yes
Xuhui	New CPV-2a	New CPV-2a	CPV-2c	CPV-2c	Yes
Baoshan	New CPV-2a	New CPV-2a	CPV-2c	CPV-2c	Yes
Qingpu	-	New CPV-2a	CPV-2c	CPV-2c	Yes
Jiading	New CPV-2a	New CPV-2a	-	-	N/A (no isolates)
Jing’an	-	-	CPV-2c	CPV-2c	Yes

## Data Availability

The original contributions presented in this study are included in the article. Further inquiries can be directed to the corresponding author.
